# BioCoder: a benchmark for bioinformatics code generation with large language models

**DOI:** 10.1093/bioinformatics/btae230

**Published:** 2024-06-28

**Authors:** Xiangru Tang, Bill Qian, Rick Gao, Jiakang Chen, Xinyun Chen, Mark B Gerstein

**Affiliations:** Department of Computer Science, Yale University, New Haven, CT 06520, United States; Department of Computer Science, Yale University, New Haven, CT 06520, United States; Department of Computer Science, Yale University, New Haven, CT 06520, United States; Department of Computer Science, Yale University, New Haven, CT 06520, United States; Google Deepmind, Mountain View, CA 94043, United States; Department of Computer Science, Yale University, New Haven, CT 06520, United States; Program in Computational Biology & Bioinformatics, Yale University, New Haven, CT 06520, United States; Department of Molecular Biophysics & Biochemistry, Yale University, New Haven, CT 06520, United States; Department of Statistics & Data Science, Yale University, New Haven, CT 06520, United States; Department of Biomedical Informatics & Data Science, Yale University, New Haven, CT 06520, United States

## Abstract

**Summary:**

Pretrained large language models (LLMs) have significantly improved code generation. As these models scale up, there is an increasing need for the output to handle more intricate tasks and to be appropriately specialized to particular domains. Here, we target bioinformatics due to the amount of domain knowledge, algorithms, and data operations this discipline requires. We present BioCoder, a benchmark developed to evaluate LLMs in generating bioinformatics-specific code. BioCoder spans much of the field, covering cross-file dependencies, class declarations, and global variables. It incorporates 1026 Python functions and 1243 Java methods extracted from GitHub, along with 253 examples from the Rosalind Project, all pertaining to bioinformatics. Using topic modeling, we show that the overall coverage of the included code is representative of the full spectrum of bioinformatics calculations. BioCoder incorporates a fuzz-testing framework for evaluation. We have applied it to evaluate various models including InCoder, CodeGen, CodeGen2, SantaCoder, StarCoder, StarCoder+, InstructCodeT5+, GPT-3.5, and GPT-4. Furthermore, we fine-tuned one model (StarCoder), demonstrating that our training dataset can enhance the performance on our testing benchmark (by >15% in terms of Pass@K under certain prompt configurations and always >3%). The results highlight two key aspects of successful models: (i) Successful models accommodate a long prompt (>2600 tokens) with full context, including functional dependencies. (ii) They contain domain-specific knowledge of bioinformatics, beyond just general coding capability. This is evident from the performance gain of GPT-3.5/4 compared to the smaller models on our benchmark (50% versus up to 25%).

**Availability and implementation:**

All datasets, benchmark, Docker images, and scripts required for testing are available at: https://github.com/gersteinlab/biocoder and https://biocoder-benchmark.github.io/.

## 1 Introduction

Large language models (LLMs) have demonstrated great success in code generation (Chen *et al.* 2021, 2023, Chowdhery *et al.* 2022, Barke *et al.* 2023, Li *et al.* 2023). The landscape of existing coding benchmarks for LLMs is largely populated with simple functions, often limited to a handful of lines (Austin *et al.* 2021, Chen *et al.* 2021, Du *et al.* 2023, Wong *et al.* 2023). Combined with a significant lack of closed-domain datasets across diverse fields, this landscape highlights the need for a more robust benchmarking system. Although domain-specific datasets, such as DS1000 (Lai *et al.* 2022) for data science, have emerged, they fall short of adequately addressing specific tasks in fields like bioinformatics. Open-domain alternatives, including HumanEval (Chen *et al.* 2021), MBPP (Austin *et al.* 2021), and APPS (Hendrycks *et al.* 2021), offer entry-level programming tasks, but their utility is limited as they lack the ability to test more niche, domain-specific code blocks. This shortfall is largely due to a lack of appropriate fine-tuning and context (Muennighoff *et al.* 2023b). Therefore, a more comprehensive approach to benchmarking is clearly needed.

**Figure 1.**
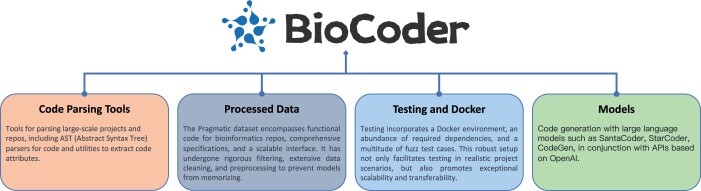
Overview of the contributions of BioCoder

To address these limitations, we introduce BioCoder (see Fig. 1), a benchmark for code generation incorporating 2269 bioinformatics-specific coding problems. Our BioCoder benchmark mainly targets bioinformatics data analysis, which tasks such as managing various biological data formats, understanding processing workflows, and utilizing APIs of various packages. This domain encapsulates the majority of daily tasks encountered by bioinformaticians in data analysis. However, BioCoder also touches upon aspects of writing bioinformatics software, particularly when tool development intersects with data analysis. Further expanding the scope of BioCoder, we included an additional 253 questions from the Rosalind project. This project specializes in generating Python functions that address key bioinformatics topics such as genetic sequencing and DNA/RNA analysis. BioCoder assures the inclusion of all potential external packages and code that could be utilized by the generated program. This consideration extends to recognizing that real-world functions often necessitate managing multiple external function calls and using global variables; hence, we included all potentially required class declarations in the input. Lastly, we performed ablation studies to determine whether the models are strictly memorizing the solutions rather than being proficient at generating code (see Supplementary Appendix M).

The key highlights of our work can be outlined as follows: (i) We create a new high-quality dataset for code generation, curated from 1720 bioinformatics repositories referenced in peer-reviewed bioinformatics articles. We processed the data, rephrasing more detailed text descriptions, as well as associated comments and specifications, including considerations needed in coding. (ii) We provide an extendable parsing tool capable of extracting all pertinent information associated with the target function in expansive projects. (iii) We provide a library for code LLMs, similar to Bui *et al.* (2023), for both training and inference in code generation tasks. (iv) We provide a fuzz testing tool capable of scaling to handle substantial datasets. Our benchmark results, derived from 1000 iterations, indicate the Pass@K rate.

## 2 Related work

Bio Coder is a code generation benchmark designed for challenging, practical bioinformatics scenarios, offering an extensible testing framework for evaluating the performance of LLMs. We provide a brief overview of the related work in both code generation models and benchmarks.

### 2.1 Code generation with LLMs

LLMs have demonstrated remarkable performances across various domains (Askell *et al.* 2021, Bai *et al.* 2022, Bommasani *et al.* 2022, Gao *et al.* 2022, Biderman *et al.* 2023, Patil *et al.* 2023, Qin *et al.* 2023, Xu *et al.* 2023). Furthermore, LLMs trained with code data have shown promising results in code, exhibiting impressive zero-shot performance on several benchmarks (Wang *et al.* 2021, Allal *et al.* 2023, Fried *et al.* 2023, Li *et al.* 2023, Olausson *et al.* 2023, Zhang *et al.* 2023b). A proven strategy to improve model performance involves increasing both the model parameters and the volume of training data (Radford *et al.* 2019, Brown *et al.* 2020, Mitchell *et al.* 2023), and many large-scale LLMs have been developed to support this endeavor (Chowdhery *et al.* 2022, Hoffmann *et al.* 2022, Thoppilan *et al.* 2022). These models have demonstrated their prowess in code generation (Brown *et al.* 2020, Chen *et al.* 2021, OpenAI 2023), and the field has also seen the release of several open-source code LLMs, such as bilingual GLM-130B (Zeng *et al.* 2022), CodeGeeX-13B (Zheng *et al.* 2023), OctoPack (Muennighoff *et al.* 2023a), WizardCoder (Luo *et al.* 2023), SantaCoder (Allal *et al.* 2023), and StarCoder (Li *et al.* 2023). Salesforce’s CodeGen (Nijkamp *et al.* 2023a,b), Huawei’s PanguCoder (Christopoulou *et al.* 2022, Shen *et al.* 2023), Meta’s LLaMA (Touvron *et al.* 2023), and CMUs InCoder model (Fried *et al.* 2023) also contribute to the field. To integrate code LLMs into real scenarios, researchers have explored methods to incorporate dependencies of relevant code in the prompt (Shrivastava *et al.* 2023).

### 2.2 Code generation datasets and benchmarks

Early work on code generation benchmarks used lexical exact match, data flow, and abstract syntax tree (AST) methods. However, these measures proved to be unreliable due to their sensitivity to inconsequential differences in the generated code. In response, execution-based evaluation approaches have become more prevalent (Chen *et al.* 2021, Khlaaf *et al.* 2022, Lai *et al.* 2022, Li *et al.* 2022, Wang *et al.* 2022b, Athiwaratkun *et al.* 2023). These approaches execute tests on the generated code to verify its functional correctness, ensuring unbiased evaluations irrespective of implementation method or style variations.

As a result, the field of code generation has seen a burgeoning number of execution-based benchmarks (Table 1) (Lee *et al.* 2023, Pan *et al.* 2023, Wong *et al.* 2023, Yuan *et al.* 2023, Zan *et al.* 2023), each presenting unique properties in terms of size, language coverage (Orlanski *et al.* 2023), complexity (Du *et al.* 2023, Zhuo 2023), and practical applicability (Yu *et al.* 2023). For instance, HumanEval (Chen *et al.* 2021) and MBPP (Austin *et al.* 2021) are frequently used code generation benchmarks that consist of 164 and 974 simple Python functions, respectively, representing a small sample size. These benchmarks also overlook the multi-language coding scenarios gap, which is partially bridged by benchmarks like HumanEval-X (Zheng *et al.* 2023) and MCoNaLa (Wang *et al.* 2023b). For a more comprehensive survey on the previous benchmarks of code generation, refer to Zan *et al.* (2023).

**Table 1.** Comparison of the statistics of BioCoder to previous benchmarks.

Benchmark	Num	Language	Data statistics					Scenario
			Test	P.C.	P.L.	C.C.	C.L.	
HumanEval (2021)	164	Python	7.8	450.6	13.7	180.9	6.8	Code Exercise
MBPP (2021)	974	Python	3.1	78.6	1.0	181.1	6.7	Code Exercise
APPS (2021)	5000	Python	21.0	1743.4	41.6	473.8	21.4	Competitions
DS-1000 (2022)	1000	Python	1.6	879.1	31.6	137.4	5.0	Data Science
HumanEval-X (2023)	164*	Multi.	7.8	468.4	15.5	264.6	12.1	Multilingual
NumpyEval (2022b)	101	Python	3.5	222.9	7.0	29.9	1.1	Public Library
TorchDataEval (2022a)	50	Python	1.1	329.0	8.6	50.7	1.3	Private Library
BioCoder (public set)	460	Multi.	1000	10 465.6	243.5	706.8	26.2	Bioinformatics
BioCoder (hidden set)	2269	Multi.	1000	12 296.7	298.8	919.5	26.2	Bioinformatics
BioCoder (similar set)	460	Multi.	1000	9885.6	240.8	767.5	26.8	Bioinformatics

Num, benchmark size; Test, average amount of test cases; P.C., average number of characters in each prompt; P.L., average number of lines in each prompt; C.C., average number of characters in the original code solutions; C.L., average number of lines in the original code solutions. This table is derived from Zan *et al.* (2023). Please refer to Zan *et al.* (2023) for a more comprehensive survey.

However, all datasets discussed above share the same shortcoming of only benchmarking generic functions, rather than domain-specific ones. DS-1000 (Lai *et al.* 2022) represents a more domain-specific dataset, featuring 1000 data science workflows extracted from Python functions. Li *et al.* (2023) reported that the performance on HumanEval and MBPP benchmarks does not always align with those on the DS-1000 benchmark. This discrepancy underscores the need for benchmarks that more accurately emulate real-world, domain-specific code generation.

In addition, the context supplied greatly influences the performance of existing LLMs (Wang *et al.* 2022a). While DS-1000 includes eight packages, it fails to fully reflect a typical coding environment. This gap is partially bridged through benchmarks such as CoderEval (Yu *et al.* 2023), which incorporate some dependencies and function calls. However, these benchmarks are rudimentary in nature and consist primarily of domain-agnostic functions. As LLMs continue to evolve, we are now beginning to see repository-level benchmarks that provide a high amount of context, such as RepoBench (Liu *et al.* 2023). However, these benchmarks remain new and untried.

Our work shares common aspects with CoderEval in its ability to evaluate models beyond the simple generation of standalone functions. Both methodologies employ Docker-based testing to handle the necessity of context-dependent code. However, our approach distinguishes itself from CoderEval by its specific emphasis on bioinformatics. We ensure that each function in our dataset requires a certain level of domain expertise in bioinformatics through a combination of automatic filtering, GPT-assisted filtering, and manual inspection. Furthermore, our dataset surpasses the scale of CoderEval, which consists of 230 functions from 43 Python projects and 230 methods from ten Java projects. In contrast, we source 2522 functions from over 2000 repositories, providing a more extensive and challenging context for code generation tasks. A comprehensive comparison between our benchmark and CoderEval can be found in Supplementary Appendix G.

## 3 The BioCoder benchmark

### 3.1 Initial dataset filtering to a set of 28 repositories

**Figure 2.**
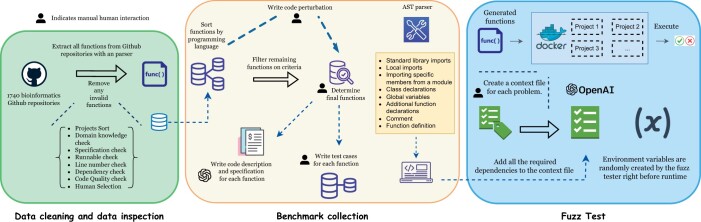
A diagram of the BioCoder construction process involving custom GitHub repository cleaning, parsing, and function selection, along with context and test case creation and a massively dockerized testing framework

Our dataset originates from an initial web scrape of 1743 bioinformatics-related GitHub repositories (see Fig. 2). Specifically, we utilized the list of 1743 bioinformatics-adjacent repositories from Russell *et al.* (2018) as the foundation for BioCoder. This list contains a curated selection of 1720 bioinformatics repositories sourced from the literature. The collected repositories include code written in various programming languages such as C, C++, PHP, Python, R, Ruby, SQL, Perl, Java, Matlab, and C#. However, for the scope of this study, we focus on Python and Java, with the intention to expand to other languages in the future. The decision to prioritize Java and Python was based on an empirical investigation into the prevalence of different programming languages across bioinformatics repositories. A more detailed discussion of this language selection process can be found in Supplementary Appendix P.

The repositories were then filtered based on popularity, community ratings, and a manual review process. This resulted in a set of 28 high-quality, highly domain-specific repositories commonly used in the field of bioinformatics. After determining this set of repositories, we developed custom Python and Java parsers to automatically analyze the selected GitHub repositories. These parsers generated an AST for each code file in the repositories and extracted relevant data, including function content, function signatures, important imports, and cross-file dependencies for each function within the code files. Upon parsing all the repositories, we obtained a large set of over 20 000 Python functions and more than 50 000 Java functions. Given this extensive baseline of functions, we conducted two rounds of automatic filtering, resulting in a final count of 1026 Python functions and 1243 Java functions (Table 2).

**Table 2.** Summary statistics for the BioCoder dataset.

	Public			Hidden			Similar		
	Py	Java	Overall	Py	Java	Overall	Py	Java	Overall
**Avg. comment lines**	4.96	2.66	4.40	8.77	4.90	6.65	5.75	3.14	5.12
**Avg. tokens of G.T.**	189.25	106.54	169.28	353.67	107.88	219.02	216.62	100.92	188.68
**Avg. lines of G.T.**	24.30	11.10	21.11	43.28	12.19	26.25	26.50	10.32	22.59
**Avg. parameters of G.T.**	2.39	1.70	2.23	2.92	1.25	2.00	2.48	1.10	2.15
**Avg. classes/function Decl.**	20.25	2.52	15.97	19.45	32.96	26.85	20.20	1.16	15.60
**Avg. global variables**	1.90	-	-	2.26	-	-	1.87	-	-
**Avg. imports**	11.91	1.52	9.40	10.37	5.00	7.43	11.63	1.16	9.10
**Avg. function calls**	7.26	4.56	6.61	14.39	6.47	10.05	9.47	5.92	8.61

**G.T.,** ground truth function; Public data, datasets with test cases; Hidden data, encompasses a wider array of intricate issues; Similar data, subset of the hidden data, mimicking the distribution of the public data (Supplementary Appendix T).

### 3.2 Topic distribution in the selected repositories

To gain an understanding of the distribution of bioinformatics within our set of 28 repositories, we applied latent Dirichlet allocation (LDA) to the abstracts of articles citing each repository. Each of these selected repositories contains the codebase associated with a single bioinformatics journal article. We used LDA to infer topics for the abstracts of articles citing each repository in the main dataset. Specifically, from the LDA model, we identified terms that were primarily associated with a single topic. We chose a model with eight topics due to its maximal coherence of concepts within the top topic-specialized terms. Finally, these eight topics were then manually labeled to summarize the top terms, resulting in the following categories: (i) Cancer and epigenetics, (ii) Proteomics and microscopy, (iii) Variant calling, (iv) Genetics and population analysis, (v) Structure and molecular interaction, (vi) Web and graphical applications, (vii) Assembly and sequence analysis, and (viii) Transcription and RNA sequencing. A detailed description of each topic can be found in Supplementary Appendix N. Our function topic filtering process can be found in Supplementary Appendix V.

### 3.3 Filtering the repositories to a list of core functions

To further filter and find a small set of functions, we started with a large baseline of functions—i.e. all the functions in the 28 repositories above—and initiated two rounds of automatic filtering to reduce the manual workload. The first round involved keyword filtering, where each function and its comments required at least 10 matches with bioinformatics-related keywords scraped from Wikipedia articles, as mentioned earlier. The methodology for obtaining this Wikipedia-based wordlist can be found in Supplementary Appendix V. Subsequently, we performed a second round of filtering, during which the OpenAI GPT-3.5 model assessed the bioinformatics relevance of each function. Finally, we manually sorted through the remaining functions, resulting in 1026 Python functions and 1243 Java functions (see Table 2). The “similar data” set in Table 2 includes an additional 157 Python functions and 50 Java functions, maintaining the same 253 Rosalind function count, reflecting the composition of the public data. These additional functions were selected to closely align with the same statistics of the public data, such as the distribution of comment lines and token counts.

Our function selection process aimed to strike a balance, ensuring that the final dataset comprises truly bioinformatics-focused functions applicable to our study. This filtering process was undertaken by experts with knowledge in bioinformatics, highlighting the essential role of bioinformatics understanding in this work.

Although our benchmark for code generation is general in nature, it is rooted in the context of bioinformatics, utilizing curated and filtered datasets based on bioinformatics problems (see Supplementary Appendix N for more details on the topic modeling and statistics regarding the overall topic coverage of the dataset). While an understanding of bioinformatics and biology may not be essential for using the benchmark, it was built to reflect the complexity and domain specifics of bioinformatics.

### 3.4 Benchmark construction

#### 3.4.1 BioCoder-Py and BioCoder-Java

**Figure 3.**
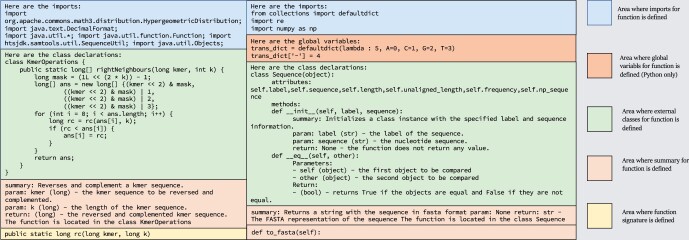
Sample prompts for code generation. Our prompts follow the same general outline. First, imports are declared at the top of the prompt, then global variables (if any), followed by function declarations, class dependencies, and finally, our actual instructions regarding the function to be generated

**Figure 4.**
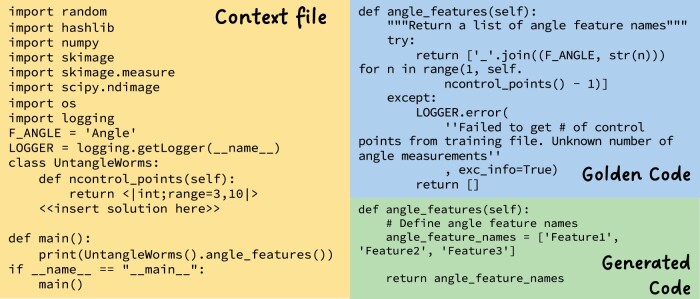
Test case for UntangleWorms example. The context file includes various import dependencies and a class definition with a method placeholder for the solution. The UntangleWorms class comes from a GitHub repository file (https://github.com/CellProfiler/CellProfiler/blob/master/cellprofiler/modules/untangleworms.py) that was scraped in our study. UntangleWorms is an image analysis tool that was initially part of the paper “An image analysis toolbox for high-throughput *C.elegans* assays”

For each function that passed all rounds of filtering described in Section 3.1, we manually wrote custom code context, including necessary imports, cross-file dependencies, and relevant fuzz test cases (detailed in Section 3.6). We then created custom prompts based on the parsed function data and summaries, ensuring the inclusion of required imports and cross-file dependencies (see Fig. 4). As we are testing function-level code generation, imports and classes are predefined and included in the context. We are not prompting the model to generate the classes needed to pass the tests, but rather testing its ability to extract pertinent imports and classes from the context for use in the generated function. Table 3 provides prompt statistics. Finally, we presented the model with a prompt to generate the function, offering the function signature as a starting point. Supplementary Appendices B and H contain examples of different prompt types. Prompts were partially generated using GPT-3.5, which was used to create function summaries for all functions in the public dataset. These summaries were incorporated into the prompts to efficiently describe the functions. Supplementary Appendix E provides more details on this method. Figure 3 shows two examples of the resulting prompt.

**Table 3.** Prompt token distribution.

Prompt	Mean	Median	STDev
Java	2278.82	2599.00	1331.81
Python	2790.75	2194.00	2539.79
Rosalind	564.49	509.00	286.47
Overall	1510.66	812.50	1882.80

#### 3.4.2 BioCoder-Rosalind

To compile the Rosalind portion of the benchmark, we began by scraping the problem descriptions from the Rosalind website, identifying problems with available solutions, and gathering all possible solutions. Subsequently, we developed a custom scraper to assemble ten test cases for each Rosalind problem. Using these test cases, we crafted a script to automatically assess whether the available solutions were successfully executed against the collected test cases.

Solutions that successfully executed against all test cases formed the “golden code” section of the Rosalind benchmark, producing correct outputs when run with the test cases. Each Rosalind benchmark context is custom-made, incorporating the scraped test cases and injecting them into the generated code. The prompts for the Rosalind problems are constructed using the scraped problem descriptions, supplemented with a brief section outlining the context into which the generated code would be integrated. This rigorous filtering process resulted in 253 functions meeting all our criteria. Selected examples for the Rosalind dataset are shown in Supplementary Appendix C. Statistics of token counts, comment lines per function, and parameters per function can be found in Supplementary Appendix A.

### 3.5 Metric

We used the Pass@K metric to measure the functional accuracy (Chen *et al.* 2021, 2022, Cassano *et al.* 2023) of code generation models. This metric quantifies, for a certain value *K*, the probability that the model can solve a particular programming problem when generating *K* candidate solutions. A problem is deemed “solved” if at least one of the *K* generated code samples passes all the test cases. Erepresents the numerical estimation for a particular problem. Each code sample represents a complete function or program intended to solve the problem. The mathematical estimation of Pass@K for a particular problem is articulated as follows:


$$\text{Pass}@K≔\underset{\text{Problems}}{E}\;\left[1-\frac{\left(\begin{array}{c} n-c\\ k \end{array}\right)}{\left(\begin{array}{c} n\\ k \end{array}\right)}\right],$$

where *n* is the number of samples generated by the model, *c* is the number of samples that pass all test cases, and *K* is the number of samples considered for the Pass@K evaluation (Chen *et al.* 2021).

### 3.6 Testing framework

Our testing framework begins with a manual review of selected functions, followed by the creation of a context file and golden code for each problem (see Fig. 4 for an example), as discussed in 3.4.

Our testing strategy is a hybrid of unit testing and fuzz testing methods, which shares similarities with the metamorphic testing methodology described in Chen *et al.* (2009). In metamorphic testing, both a reference implementation and the test code are provided with parametrically generated input data to ensure identical behavior. While our approach is not strictly metamorphic testing, it leverages similar principles by using a golden code as a reference and generating random test inputs to compare outputs.

For Python and Java functions, we use a custom syntax in the context file to indicate insertion points for randomly generated test cases, representing four data types: integers, floats, strings, and Boolean values. During runtime, these insertion points are replaced with language-specific code to insert dynamically generated test cases. The tester can be run for any number of iterations, depending on the desired number of fuzz tests.

For Rosalind functions, the process is simpler and more efficient as the functions are less complex. The output of the golden code is generated and cached ahead of time. During testing, the tester executes the generated code within the corresponding context and compares the output with the cached golden code output.

We ran the golden output against itself for every fuzz and Rosalind test case to ensure 100% reliability. To ensure system security and test reliability, we ran our tests in Docker environments using Amazon Web Services, coordinating tasks across multiple nodes to accelerate the process without compromising result validity. After creating a generalized Docker image with all necessary Python requirements, we summarized our testing framework in Supplementary Appendix K and addressed potential concerns about testing issues due to package changes in Supplementary Appendix S.

## 4 Models and results

To test BioCoder, we opted to benchmark StarCoder-15B (Li *et al.* 2023), StarCoder+-15B (Li *et al.* 2023), InCoder (Fried *et al.* 2023), SantaCoder (Allal *et al.* 2023), CodeGen (6B-mono and 16B-mono) (Nijkamp *et al.* 2023b), CodeGen2-7B (Nijkamp *et al.* 2023a), InstructCodeT5+ (Wang *et al.* 2023a), and GPT3.5-Turbo and GPT-4 (OpenAI 2023) through Azure OpenAI Service. Full details of the model context lengths and model sizes can be found in Table 4.

**Table 4.** Context length limits and sizes of different code LLMs.

Model	Context limit	No. of parameters
InCoder (Fried *et al.* 2023)	2048	6B
SantaCoder (Allal *et al.* 2023)	2048	1.1B
StarCoder (Li *et al.* 2023)	8192	15.5B
StarCoderPlus (Li *et al.* 2023)	8192	15.5B
InstructCodeT5+ (Wang *et al.* 2023a)	2048	16B
CodeGen-6B (Nijkamp *et al.* 2023b)	2048	6B
CodeGen-16B (Nijkamp *et al.* 2023b)	2048	16B
CodeGen2 (Nijkamp *et al.* 2023a)	2048	7B*
GPT-3.5-Turbo	8192	Unknown
GPT-4	8192	Unknown

To target specific performance characteristics, we came up with hundreds of variations of the prompt. We chose three goals: test the performance of models with extraneous context, without extraneous context, and any context. These goals allow us to better analyze failure reasons and the effectiveness of our context-driven approach. After careful experimentation, we settled on the prompt type shown in Fig. 3, which we call *Summary at Bottom*. Following the instruction paradigm of some considered models, we test a version with the summary moved to the top, along with the text “# Here is an instruction. Complete the function using the required context.” To test without extraneous context, we used human annotators to manually determine the required context and used the structure of the *Summary at Top* prompt. Further prompt explanations can be found in Supplementary Appendix H.

Below is an explanation of the prompt types:

1. *Summary Only*: These prompts only contain the summary and the function signature, with the uncommented summary coming before the signature. Note that the summary includes nearly complete details about the task; however, it intentionally does not thoroughly explain what the context is. Therefore, this result is best treated as a baseline when compared with other prompt types.
2. *Uncommented*: These prompts contain the full parsed context (including the imports, global variables classes, internal class functions, etc), the summary, and the function signature, in that order. For functions exceeding ten lines in the context, we summarize the parameters, return type, and purpose instead of including the full function code. This step streamlines the number of input tokens and eliminates extraneous data.
3. *Summary at Bottom*: These prompts have the same structure as the uncommented ones, but we add the context as a comment. In addition, there are no results for “summary at bottom” for Java due to incompatibility with Java syntax. We were unable to generate this type of prompt for Java in a similar manner to how we generated it for Python.
4. *Summary at Top:* These prompts contain the summary, the full (commented) parsed context, and the function signature, in that order. For Java, the summary is not copied at the bottom. This is intended for models with shorter context lengths, as when we truncated the prompt (usually only affecting the context), the summary would still be intact, along with a portion of the context.
5. *Necessary Only:* We use a mixture of our syntax-solving algorithm and hand annotation to select precisely which objects within the context are necessary for the function to execute. Note that this is very similar to the environment used for testing the functions.

To accurately represent the performance of the LLM outputs, we implemented basic correction mechanisms to rectify minor syntax and style errors that did not impact functionality. For instance, all StarCoder outputs were appended with a postscript. Each LLM output was then passed through these correction mechanisms before being sent to the testing framework for evaluation (see Tables 5 and 6).

**Table 5.** Zero-shot and fine-tuned performance with five prompt versions of BioCoder.

Model	Prompt	Java				Python			
		Pass@1	Pass@5	Pass@10	Pass@20	Pass@1	Pass@5	Pass@10	Pass@20
InCoder-6B	*Summary at Top*	0	0	0	0	0.828	2.016	3.006	4.459
	*Uncommented*	0	0	0	0	0.032	0.159	0.318	0.637
	*Summary Only*	0	0	0	0	1.688	5.320	8.332	12.006
	*Necessary Only*	0	0	0	0	0.032	0.159	0.318	0.637
SantaCoder-1.1B	*Summary at Top*	0	0	0	0	0.637	1.338	1.844	2.548
	*Uncommented*	0	0	0	0	0.287	0.764	0.955	1.274
	*Summary Only*	0	0	0	0	2.965	9.848	14.227	18.181
	*Necessary Only*	0	0	0	0	0.032	0.159	0.318	0.637
StarCoder-15.5B	*Summary at Top*	0	0	0	0	3.694	13.197	19.359	24.554
	*Uncommented*	0	0	0	0	0.318	1.062	1.591	2.548
	*Summary Only*	0	0	0	0	4.682	15.225	21.200	27.166
	*Necessary Only*	0	0	0	0	0.127	0.603	1.123	1.911
StarCoder-15.5B (finetuned)	*Summary at top*	0	0	0	0	5.818	16.562	21.091	27.048
	*Uncommented*	0	0	0	0	3.312	9.073	12.574	17.536
	*Summary Only*	0.200	1.000	2.000	4.000	7.295	20.838	26.143	39.570
	*Necessary Only*	3.300	12.097	19.545	30.000	0.597	1.173	1.813	2.611
StarCoder+	*Summary at Top*	0	0	0	0	2.675	9.133	14.019	19.650
	*Uncommented*	0	0	0	0	0.510	0.955	1.274	1.911
	*Summary Only*	1.300	5.031	8.042	12.000	2.548	8.279	12.864	18.057
	*Necessary Only*	0	0	0	0	0.127	0.457	0.609	0.637
InstructCodeT5+	*All prompt types*	0	0	0	0	0	0	0	0
CodeGen-6B-mono	*Summary at Top*	0	0	0	0	0.637	0.637	0.637	0.637
	*Uncommented*	0	0	0	0	0	0	0	0
	*Summary Only*	0	0	0	0	0.637	0.637	0.637	0.637
	*Necessary Only*	0	0	0	0	0	0	0	0
CodeGen-16B-mono	*Summary at Top*	0	0	0	0	0.637	0.637	0.637	0.637
	*Uncommented*	0	0	0	0	0	0	0	0
	*Summary Only*	0	0	0	0	0.637	0.637	0.637	0.637
	*Necessary Only*	0	0	0	0	0	0	0	0
CodeGen2-7B	*Summary at Top*	0	0	0	0	0.637	0.637	0.637	0.637
	*Uncommented*	0	0	0	0	0.510	0.637	0.637	0.637
	*Summary Only*	0	0	0	0	0.860	2.494	3.962	6.242
	*Necessary Only*	0	0	0	0	0	0	0	0
GPT-3.5-Turbo	*Summary at Top*	4.100	7.235	8.989	11.600	22.771	33.461	36.551	39.490
	*Uncommented*	6.300	11.563	14.436	18.000	11.019	19.075	21.680	24.204
	*Summary Only*	17.400	33.199	37.878	42.000	24.682	33.997	37.132	40.127
	*Necessary Only*	43.500	52.582	53.995	55.400	28.758	39.529	44.029	47.771
GPT-4	*Summary at top*	1.100	5.500	11.000	22.000	10.701	25.500	32.910	39.490
	*Uncommented*	6.367	11.234	15.897	18.562	12.654	20.129	24.387	27.932
	*Summary Only*	19.483	24.721	29.634	2.543	13.172	24.578	28.394	31.938
	*Necessary Only*	**45.011**	**55.350**	**57.616**	**60.000**	**38.439**	**48.491**	**50.619**	**52.229**

For examples of each prompt version, see Supplementary Appendix H. For all settings, we performed trials twice for Pass@K. Results are expressed in percentages. We only fine-tuned StarCoder for 2000 steps; all others are zero-shot results. Additional results can be found in Supplementary Appendix Q (*Summary at Bottom* results are omitted here).

Bold values refer to the models with the best performance.

**Table 6.** Performance on Rosalind.

Model	Prompt	Rosalind			
		Pass@1	Pass@5	Pass@10	Pass@20
InCoder	*Description*	0.020	0.099	0.198	0.395
SantaCoder	*Description*	0.158	0.658	1.075	1.581
StarCoder	*Description*	0.534	2.042	3.228	4.743
StarCoderPlus	*Description*	0.356	1.313	1.978	2.767
StarCoder (fine-tuned)	*Description*	1.623	3.109	5.328	7.036
InstructCodeT5+	*Description*	0.059	0.296	0.593	1.186
CodeGen	*Description*	0.692	2.088	3.055	3.953
CodeGen2	*Description*	0.059	0.296	0.593	1.186
GPT-3.5 Turbo	*Description*	23.671	31.953	36.702	40.725
GPT-4	*Description*	**24.308**	**39.551**	**44.864**	**50.198**

In this table, we have omitted the percentage symbol (%), although these figures represent the Pass@K in the form of percentages. For all settings, *n* = 20.

Bold values refer to the models with the best performance.

Furthermore, to empirically evaluate the hypothesis regarding the efficacy of smaller, specialized LLMs in closed-domain code generation, as opposed to large open-domain pretrained models like GPT-3.5 and GPT-4, we fine-tuned StarCoder and documented the resulting performance. We chose StarCoder as a representative sample of currently popular models. Due to computing constraints, we were unable to fine-tune all the models, but we encourage contributions from the broader community. Inference was executed on HPC clusters equipped with 8× A100 GPUs.

The results in Tables 5 and 6 align with our initial hypothesis, which proposed that larger models would likely outperform their smaller counterparts. However, the significant performance gap between GPT-3.5, GPT-4, and all other code-generation models was surprising. This underscores the crucial role of both the dataset size and parameter size of the base models in accomplishing closed-domain code generation prompts. Java performance improved significantly, as the structure is similar between the training set and testing set. Interestingly, despite the rudimentary nature of our fine-tuning on StarCoder, the results still highlighted a significant improvement compared with the nonfine-tuned model. This stark contrast in performance bolsters our original assertion: achieving success in closed-domain tasks can be realized either through large open-domain LLMs or via fine-tuning smaller models. These smaller models could potentially achieve comparable performance but with significantly reduced computational and memory requirements. Furthermore, Table 5 demonstrates that the performance of models improves with the inclusion of dependencies in prompts. Without additional training, ChatGPT models performed notably better than other models. Their performance underscores the crucial role of both the dataset scale and model size. That said, the performance of other models (e.g. StarCoder) could be improved through fine-tuning.

## 5 Analysis and discussion

Looking more closely at the results in Table 5, it is clear that the larger models with more parameters generally perform better than the smaller models. The GPT-4 model dwarfs the other models in this study in both size and performance. However, it is clear that BioCoder remains a challenge as GPT-3.5 and GPT-4, the best models, only achieved an accuracy of slightly under 60%.

Examining the other models, it is interesting to note that while InstructCodeT5+, CodeGen, and CodeGen2 are all larger than InCoder and SantaCoder, they perform far worse. This is likely due to the former being trained for single-line completions rather than function completion. Furthermore, InstructCodeT5+, CodeGen, and CodeGen2 have relatively small context limits (Mikolov *et al.* 2013), which likely hurts their performance. As for the remaining model, SantaCoder notably performs impressively well for being only a roughly 1B parameter model, which is an indication of aggressive fine-tuning on Python code.

We also note that the context length limit has a substantial impact on how different models perform on different prompts. Except for GPT-3.5 and GPT-4, models performed the best on the *Summary Only* prompt style, likely because of its shorter length. Summary-only prompts are shortened prompts utilized across all our LLM models to ensure that context-limited LLMs still receive all the necessary information necessary to potentially generate functions. Within the summary-only prompts, we optimized our prompts to contain only the absolute minimum of necessary information, without including much of the additional context that provides detail regarding the functionality of other dependencies. Looking at Fig. 3, which contains the complete prompt structure, summary-only prompts would reduce the class declarations to only their declarations and one sentence describing their output and input. This is especially pronounced for InCoder and SantaCoder, as they both have small context limits of 2048 tokens. Their Pass@K performance for Python decreases dramatically when switching from short *Summary Only* prompts to longer *Summary at Top/Bottom* ones.

As shown by the scatterplots in Supplementary Appendix J reveal an inverse relationship between the number of tokens in the prompt and the Pass@K score for models with an average Pass@K score of at least 2%. Furthermore, for models such as SantaCoder and GPT, the performance sharply declines after around 500 tokens. This could be due to the massive amount of context “confusing” the models. However, model performance cannot only be attributed to prompt length. We can see that even though the *Necessary Only* prompts are relatively shorter when compared to the *Summary at Top* or *Uncommented* prompts, the Pass@k performance of the “Uncommented” prompts is worse for many of the models. For further analysis of this and prompt structure in general, please refer to Supplementary Appendix U.

Focusing on Java’s performance, it is clear that most of the publicly available LLMs have not been fine-tuned for Java, resulting in near 0% Pass@K values. Finally, the performance results for Rosalind in Table 6 are roughly in line with Python’s performance in Table 5.

Table 7 provides an overview of the error statistics collected from our test runs. The errors include: “different output,” where the generated code’s output did not match the golden code’s output; “invalid syntax,” where syntax errors in the generated code prevented code execution; “function timed out,” where code execution exceeded time limits; and “runtime error,” where the generated code compiled successfully but failed to run. The vast majority of the generated code samples tested encountered a syntax or runtime error without resulting in an output. See Table 8 for more detail. Additional error statistics per model can be found in Supplementary Appendix O. Looking at Supplementary Appendix O, it appears that the models struggle the most with writing code that will successfully compile or run. For the code that did produce outputs, however, GPT-based models produced more correct samples than incorrect ones (differing output), while other models generated more incorrect but syntactically valid code. Therefore, it seems that the better-performing models have the most trouble generating syntactically correct code rather than understanding the logic required to complete the problems outlined in the prompts. Further discussion on the results of each model can be found in Supplementary Appendix I. Despite these challenges, we believe that this dataset holds importance for benchmarking future models, especially ones with larger context limits, such as GPT-4-32k and Claude2.

**Table 7.** Aggregated error distribution across all models.

Failure/Success	Count	Percent (%)
Mismatched output	8661	4.567
Invalid syntax	117 665	62.038
Runtime error	55 351	29.184
Time out	4	0.002
Successfully passed	7982	4.209
Total testcases	189 663	100

**Table 8.** Failure modes for selected (best-performing) models, corresponding to the results shown in Table 4.

Model	Prompt	Pass	Differing	Runtime Err.	Syntax Err.	Other	Model	Prompt	Pass	Differing	Runtime Err.	Syntax Err.	Other
GPT-3	*Summary at top*	756	664	1509	1207	4	GPT-4	*Summary at top*	358	119	2321	1342	0
	*Uncommented*	409	254	435	3040	2		*Uncommented*	15	9	2796	1320	0
	*Summary Only*	949	863	1528	799	1		*Summary Only*	411	356	1765	1608	0
	*Summary at bottom*	422	319	854	1545	0		*Summary at bottom*	25	2	1174	1939	0
	*Necessary Only*	1338	1103	1275	420	4		*Necessary Only*	1660	1094	996	388	2
StarCoder	*Summary at top*	116	160	1473	2390	1	Codegen2-7B	*Summary at top*	20	0	2326	1794	0
	*Uncommented*	10	18	484	3628	0		*Uncommented*	16	1	508	3612	3
	*Summary Only*	147	258	1885	1849	1		*Summary Only*	27	136	1589	2386	2
	*Summary at bottom*	203	395	857	1682	3		*Summary at bottom*	16	69	2182	873	0
	*Necessary Only*	4	9	85	4042	0		*Necessary Only*	0	5	113	4022	0

Pass, generated and golden code exhibited the same functionality; Differing, code that compiled and ran, but gave different outputs with respect to the golden code when executed; Runtime Err., code compiled but unexpectedly crashed during execution; Syntax Err, code did not compile; Other, timeouts resulting from mistakes such as infinite loops. Note that we intentionally set the execution limits to be generous, so if a generated code sample failed given our constraints, then the sample would not be used in practice anyway.

## 6 Limitations

Our study has several limitations that warrant discussion. First, the use of closed-source LLMs, such as OpenAIs GPT, may introduce a degree of circularity in our methodology. The lack of transparency regarding the training data and model architecture of these LLMs makes it challenging to determine whether the test cases used in our benchmark are truly independent of the models’ knowledge base. Despite our obfuscation step (including the ablation study in Supplementary Appendix M), this circularity could potentially lead to inflated performance metrics and hinder the generalizability of our findings. We partially mitigated this issue by including open-source models in the evaluation. Future research should prioritize the use of open-source LLMs or collaborate with LLM providers to ensure a clear separation between training and testing data.

Moreover, the closed-source nature of some LLMs used in our study raises concerns about reproducibility and fairness in performance comparisons. The constant evolution and updates to these models, often without detailed release notes, may make it difficult to replicate our findings or conduct longitudinal studies. To address this issue, we encourage the development and adoption of standardized benchmarking protocols and the use of versioned, open-source models whenever possible. This includes the usage of mechanisms such as the OpenAIs “system fingerprint” and “seed” parameters.

Lastly, by using OpenAI GPT to assist in identifying bioinformatics-related code samples, we may have favored examples that align with its preexisting knowledge or biases. This bias could result in a dataset that is more easily solved by OpenAI GPT-like models, potentially skewing the performance evaluation. Future work should explore alternative approaches to dataset curation that minimize reliance on the same type of models being evaluated.

Despite these limitations, our study serves as a valuable starting point for evaluating the performance of LLMs in bioinformatics code generation. By acknowledging and addressing the identified challenges, future research can build upon our work to develop more robust, transparent, and comprehensive benchmarking frameworks. Such efforts will be essential in advancing the responsible and effective application of LLMs in bioinformatics research and practice.

## 7 Conclusions and future work

Our study underscores the challenges in code generation, emphasizing the shortcomings of current models in the face of complex tasks. We present highly challenging natural language to code tasks, providing input rich with dependencies and imports. Existing models struggle to comprehend the application of these imported toolkits or functions contained in other files. Our tasks are marked by extensive input and a high level of specialization. These programs are closer to real-world scenarios, requiring professional-level code-writing skills, rather than merely catering to beginners. This suggests that the code in question can typically be produced only by professional programmers.

We welcome contributions to our benchmark. Our data are stored in the JSON format, with the fields documented in our GitHub repository. To ensure compatibility and ease of integration, we recommend that contributors follow the existing data structure and provide the necessary information for each new test case, such as the problem description, input/output examples, and any additional context or dependencies.

As a novel benchmark within the field of bioinformatics, there remain a multitude of areas for future exploration. While we have covered most existing models and included a few well-established repositories, future work could expand the dataset to include more niche substudies and programming languages. Additionally, our benchmark may not exhaustively cover all relevant domains and emerging techniques. As bioinformatics evolves, it is crucial to update and expand the benchmark to reflect the latest challenges and methodologies, moving beyond function-level code generation to include more complex, multi-step workflows that require planning and better simulate real-world use cases. An example of such a task for realistic assessment could involve initially interpreting a count matrix and subsequently identifying different cell types present based on the count data. These types of tasks require the model to execute multiple routines in a sequence and to adapt based on prior outcomes.

## Supplementary Material

btae230_Supplementary_Data
